# INulin-type β-fructans supplementation to modulate gut microbiota and assessment of its effects on health status and GUT-SKIN axis parameters in patients with psoriasis (INGUTSKIN): the 8-week, randomised, double-blind, placebo-controlled clinical study protocol

**DOI:** 10.1186/s12937-026-01316-8

**Published:** 2026-03-17

**Authors:** Urszula Krupa-Kozak, Agnieszka Owczarczyk-Saczonek, Ewa Lange, Małgorzata Starowicz, Marta Kopcewicz, Paulina Kęszycka, Joanna Czerwińska, Joanna Rybak-d’Obyrn, Michał Oczkowski, Julien De Biasi, Karolina Bieglecka, Maja Kleniewska, Natalia Bączek, Jadwiga Małkowska, Krzysztof Pastuszak

**Affiliations:** 1 InLife Institute of Animal Reproduction and Food Research of the Polish Academy of Sciences, Trylińskiego Street 18, Olsztyn, 10-683 Poland; 2 Department of Dermatology, Sexually Transmitted Diseases and Clinical Immunology, School of Medicine, Collegium Medicum, University of Warmia and Mazury in Olsztyn, Wojska Polskiego Street 30, Olsztyn, 10-719 Poland; 3 Department of Dietetics, Institute of Human Nutrition Sciences, Warsaw University of Life Sciences (SGGW-WULS), Nowoursynowska Street 159 C, Warsaw, 02-776 Poland; 4 Department of Algorithms and System Modeling, Gdańsk University of Technology, Gdańsk, Poland; 5 Department of Translational Oncology, Medical University of Gdańsk, Gdańsk, Poland; 6 Center of Biostatistics and Bioinformatics, Medical University of Gdańsk, Gdańsk, Poland

**Keywords:** Chronic skin inflammation, Prebiotic, Inulin-type fructans, Nutritional intervention, Quality of life, Gut microbiota, Intestinal barrier, Volatile organic compounds, Oxidative stress, Metabolic parameters

## Abstract

**Background:**

A strong, bidirectional association exists between gastrointestinal health and skin homeostasis in many chronic skin inflammations, including psoriasis (PS). It is postulated that, apart from genetic predisposition, the rise of local and systemic immune response in PS could be a consequence of intestinal dysbiosis associated with increased intestinal permeability. We hypothesised that restoring gut microbiome homeostasis and proper functioning of the intestinal barrier in PS patients would alleviate the inflammatory symptoms and severity of the skin lesions. The study aims to determine the effect of chicory-derived inulin-type β-fructans (ITFs) on health-related parameters in mild PS patients through molecular analysis of gut microbiota characteristics and assessment of a wide spectrum of biomarkers of the gut-skin axis.

**Methods:**

The randomised, placebo-controlled clinical trial (RCT) with prebiotic intervention is proposed. Adult mild PS patients (Psoriasis Area and Severity Index; PASI < 10) with a body mass index (BMI) < 30 kg/m^2^ following an omnivore diet will be enrolled into the trial and randomized to one of two groups: prebiotic (receiving 15 g/per day of ITFs) or placebo (receiving 15 g/per day of maltodextrin) for 8 weeks in a double-blind manner. Body composition, clinical parameters, nutritional status, quality of life, immune and inflammatory parameters, intestinal barrier permeability, characteristics of faecal bacteria, and metabolic dysfunction markers will be determined at baseline and after supplementation. Compliance and adverse reactions will be evaluated.

**Discussion:**

The dysregulation in intestinal microbiota-host interplay is connected with the development of many chronic skin inflammations, including PS. A proper diet is a relatively easy-modifiable factor that may influence the course of PS treatment. Among dietary components, prebiotics have garnered our interest as ingredients with a proven ability to enhance host health by modulating the gut microbiota. With the proposed RCT, we will determine the impact of ITFs on gut microbiota characteristics and evaluate whether this ITFs-induced microbiota modulation is an effective method to alleviate inflammation and reduce the severity of skin lesions in PS. We expect that the RCT results will enable the introduction of personalised dietary therapy with prebiotic ITFs, which, unlike other PS therapies, would not pose a high risk of side effects.

**Trial registration:**

ClinicalTrials.gov Registration Number: NCT05971992.

## Background

Psoriasis (PS) is a non-contagious chronic skin inflammation that occurs in approximately 2–3% of the global population in most ethnic groups [1]. It is characterised by various symptoms, including flaky skin (patches), itchiness, and redness of the affected area. In most cases (approximately 70–80%), psoriatic skin lesions are mild [2] and do not require systemic treatment or immunosuppressants, which may exert severe side effects. Nevertheless, novel and safe therapeutic approaches are still demanded to treat psoriasis lesions.

Although the pathogenesis of PS is not fully understood, it is known that the main cause of PS development is genetic predisposition [3, 4], which, in combination with environmental factors may lead to a dysregulation of the immune responses, observed as accelerated tumour necrosis factor(TN)/interleukin-23 (IL-23)/IL-17 axis, and hyperproliferation of epidermal keratinocytes.

Recently, considerable interest has focused on the interactions among the intestinal microbiota, intestinal barrier, and immune system in PS [5–7]. The studies by Sikora et al. [6] support the hypothesis that the inflammatory process in PS may disrupt the gastrointestinal barrier. In addition, recent studies indicate that the intestinal microbiome profile of PS subjects significantly differs from that of healthy subjects [8–11]. Thus, the so-called “gut-skin axis” has been considered a key factor in the aetiology of PS and a potential therapeutic target [12].

The relationship between dietary habits and the severity of psoriasis has rarely been studied. Meanwhile, epidemiological studies have shown that individuals with PS have unbalanced macronutrient intake, in particular higher intakes of simple carbohydrates and total fat, and lower intakes of protein, complex carbohydrates, dietary fibre, omega-3 polyunsaturated fatty acids (PUFAs), and vegetables [13, 14]. Diet is a relatively easy-to-modify factor that may influence the course of PS and the effectiveness of its treatment. The application of dietary fibre in the diet has been reported to show systemic and intestinal anti-inflammatory effects [15, 16]. Fibre may also promote the growth of commensal bacteria and increase the resistance to the colonisation of pathogenic bacteria, correcting the dysbiosis in gut microbiota in PS [17].

Prebiotics are dietary ingredients with great potential to influence human health beneficially through their impact on the growth and/or activity of health-promoting bacteria that colonise the gastrointestinal tract. Consumption of prebiotics resulted in a significant increase in bifidobacteria and lactobacilli and a reduction in the count of pathogenic bacteria, such as clostridia [18]. Moreover, prebiotics influence the activity of the immune system and certain immunological biomarkers, mainly due to a modulation of gut bacterial composition and activities [19–21]. Thus, prebiotics as gut microbiota-modulating agents should become a research target to alleviate inflammation and the severity of skin dermatitis.

Inulin-type fructans (ITFs) are a group of naturally occurring plant carbohydrates that are classified as prebiotics. The ITFs have many beneficial effects on inflammatory bowel diseases, including affecting the histological picture of the intestine (proliferation in the crypts and goblet cells, longer intestinal villi), changing the profile of mucins and modulating endocrine and immune functions [22]. Moreover, they have great potential as agents for improving or maintaining a balanced intestinal microbiota. Despite the benefits resulting from ITFs intake, their efficacy in managing skin inflammation is not widely documented. Thus, high-quality studies analysing the effect of prebiotics on PS subjects are urgently needed.

### Study objective

The primary aim of this original and complex randomised controlled trial (RCT) is to determine the effect of chicory-derived ITFs supplementation on health-related parameters in PS patients through molecular analysis of gut microbiota characteristics and assessment of a wide spectrum of metabolic parameters and biomarkers of the gut-skin axis. We expect that prebiotic ITFs, as a dietary supplement, will provide several health benefits to PS subjects beyond the gastrointestinal microbiota, such as neutralising systemic inflammation, reducing the severity of skin lesions, and rebuilding/stabilising the intestinal barrier, without causing side effects. Additionally, we expect the proposed RCT to determine whether the identified health-related benefits are evoked by compositional and/or functional changes of the intestinal bacterial communities.

### Hypothesis

We hypothesised that supplementation of PS subjects with prebiotic ITFs would restore gut microbiome homeostasis, improve gut barrier function, and reduce systemic inflammation, which would consequently lead to alleviation of inflammatory symptoms and a decrease in the severity of skin lesions.

## Methods

### Study design

A single-centre, randomised, placebo-controlled, double-blind clinical trial with parallel arms and equal allocation is proposed. Additionally, a control group of healthy subjects will be established. The study adheres to the Consolidated Standards of Reporting Trials (CONSORT) 2010 [23] and Standard Protocol Items: Recommendations for Interventional Trials (SPIRIT) 2013 Statements [24] (Related file). The trial has been registered at ClinicalTrials.gov (NCT05971992) on August 1, 2023.

### Participants

**Fig. 1:**
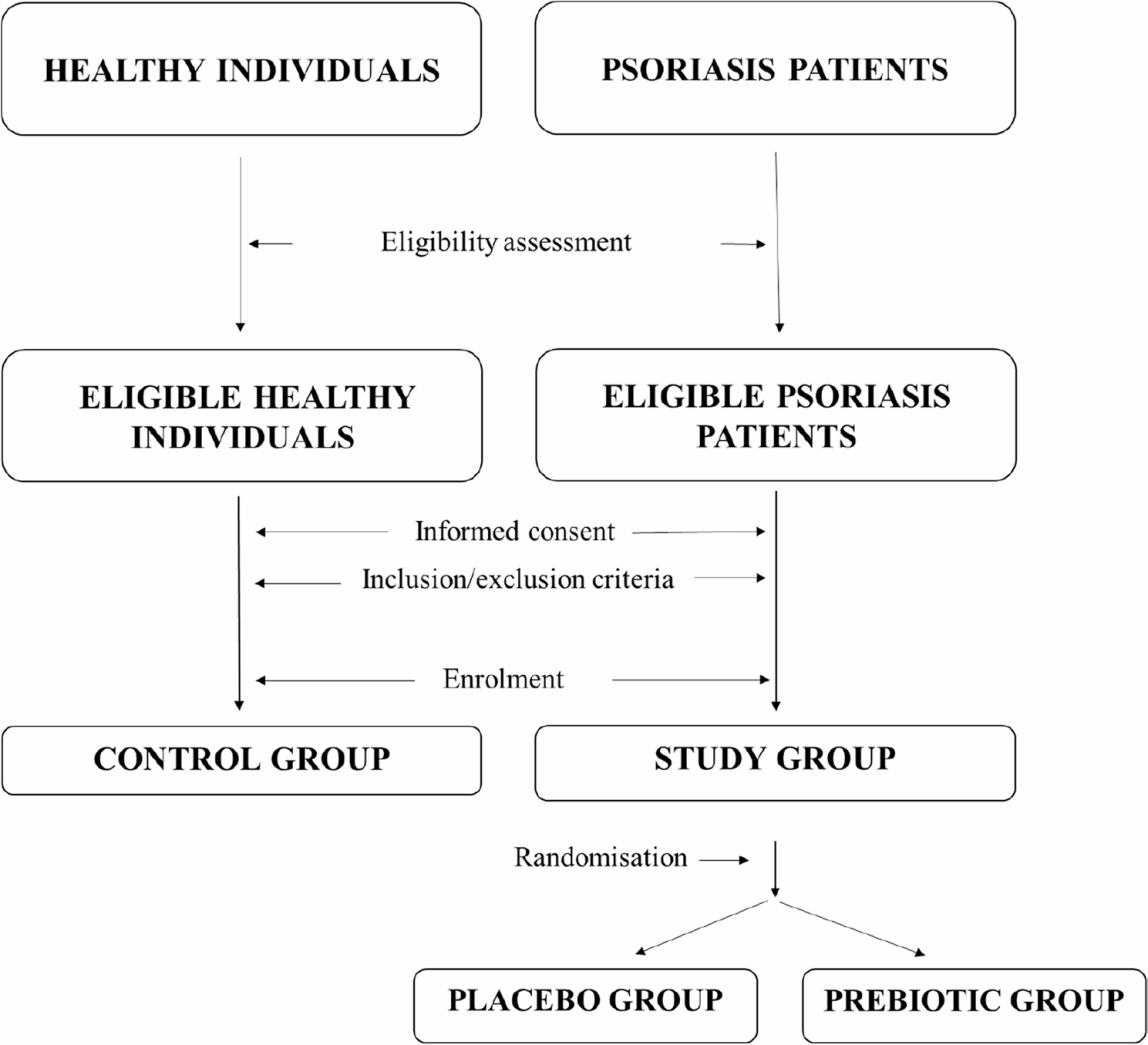
Flow diagram of participant recruitment according to CONSORT [23]

The participants will be recruited by a dermatologist among the adult patients diagnosed with PS, who are inpatients and outpatients of the Clinic and Department of Dermatology, Sexually Transmitted Diseases and Clinical Immunology of the Municipal Hospital in Olsztyn (Poland). The PS patients will be invited by phone to participate in the study voluntarily (Fig. 1). Eligible patients interested in the study will be scheduled for an enrolment visit to the Dermatology Clinic. Mild PS patients (PASI < 10) [2] who will voluntarily give their informed consent form and meet the inclusion and exclusion criteria (Table 1) will be enrolled in the study group.

Healthy individuals will be invited to participate in the study voluntarily through an advertisement posted on the Institute of Animal Reproduction and Food Science of the Polish Academy of Sciences (IAR&FR PAS) website (www.pan.olsztyn.pl). Individuals interested in the study will be scheduled for the enrolment visit at the Dermatology Clinic to sign the informed consent form. They will undergo the eligibility assessment (Table 1). Ultimately, healthy subjects who meet the inclusion and exclusion criteria will be enrolled in the control group (Fig. 1).

**Table 1:** Criteria for inclusion and exclusion

Study group	Control group
Inclusion Criteria	Inclusion Criteria
• Age 18–65 years old • Mild psoriasis (PASI < 10) • Body mass index (BMI) < 30 kg/m^2^ • Omnivorous diet, • Negative tTG-IgA, • Willingness to give informed consent	• Age 18–65 years old • Good health • Body mass index (BMI) < 30 kg/m^2^ • Omnivorous diet, • Negative tTG-IgA, • Willingness to give informed consent
Exclusion Criteria	Exclusion Criteria
• Chronic or acute inflammatory disease (other than psoriasis), in particular diabetes, gastrointestinal disease, cardiovascular complications, heart, kidney and liver failure, thyroid disease, cancer • Current anti-psoriatic treatment (systemic or biologic) • Antibiotics within 14 days before sampling • Probiotic, prebiotic, and/or synbiotic supplements intake within 4 weeks before sampling • Pregnancy, breastfeeding • Surgery within 6 months before sampling	• Chronic or acute diseases, in particular, skin disease, diabetes, gastrointestinal disease, cardiovascular complications, heart, kidney and liver failure, thyroid disease, cancer • Antibiotics within 14 days before sampling. • Probiotic, prebiotic, and/or synbiotic supplements intake within 4 weeks before sampling • Pregnancy, breastfeeding • Surgery within 6 months before sampling

### Supplements

In this single-centre, double-blind, randomised placebo-controlled trial with 8-week nutritional intervention, a prebiotic ITFs formulation (Orafti^®^Synergy1, Beneo, Tienen, Belgium), consisting of a unique combination of chicory-derived inulin fractions with selected chain lengths (shorter chain (DP 3–9) and longer chain (DP ≥ 10) inulin in essentially equal amounts), was applied. According to the International Scientific Association for Probiotics and Prebiotics (ISAPP) statement, fructans are scientifically proven prebiotics of the most extensively documented health benefits in humans [25]. Additionally, the US Food and Drug Administration has announced that chicory root fibre is an approved dietary fibre [26]. Maltodextrin (Nutridex, OMNİA EUROPE SA., Medgidia, Romania), an odourless powder with an appearance and taste similar to ITFs, was selected as the placebo supplement in this RCT. This polysaccharide, easily digested and largely absorbed in the small intestine, is a common choice in ITFs studies [27, 28]. Thus, in our RCT, we chose maltodextrin based on guidance from the literature and our previous experience [29]. Both supplements were portioned into identical-looking, moisture-impermeable sachets by the outsourcing company (EDPOL Food & Innovation Sp. z o.o., Łomża, Poland). Each sachet contained 7.5 g of the appropriate powder. Packages containing the required number of sachets, either prebiotic or placebo, were prepared for the entire intervention period to be distributed to the enrolled participants.

### Randomisation and blinding

In the present RCT, PS patients will enter the trial sequentially, and their covariates (sex, age, BMI) will be used to adapt the randomisation process. A covariate-adaptive randomisation procedure for two treatments will be applied to balance the treatment allocations across the selected covariates (sex, age, BMI) using R software. The randomisation procedure will be conducted by a biostatistician who is not directly involved in the study. At the allocation visit, PS patients, who are randomly allocated to either the prebiotic or placebo group, will obtain a package of sachets containing the supplement (prebiotic or placebo) from a researcher not directly involved in the study. The trial employs a double-blind design, in which the researchers (excluding the one responsible for supplement distribution), laboratory personnel, and participants are unaware of which subjects receive the prebiotic and which the placebo.

### Intervention

In this RCT, the nutritional intervention will last 8 weeks. During the first week, participants in the study group will be instructed to consume only one sachet of the supplement daily with breakfast to minimise potential side effects. From week 2 until the end of the intervention, participants will consume two sachets daily (at breakfast and dinner), amounting to a total dose of 15 g of supplement (prebiotic or placebo) each day. Participants will be instructed to dissolve the sachet content in water or juice and consume it 15–20 min before a meal. The PS participants will receive questionnaires on compliance and adverse reactions, and will be asked to record their supplement intake, any adverse reactions (gastrointestinal and other), and medication use during the study. The dosage of the supplement and the duration of the intervention were based on previous studies involving adults [28]. Participants from the control group (healthy individuals) will not be the object of any supplementation.

### Study outcomes

The primary outcome is the between-group difference in the change in PASI score from baseline (T0) to the close-up visit (T1) after the 8-week intervention, which will inform about the change in the severity of skin lesions. The secondary outcomes are changes within and between the prebiotic and placebo group after the 8-week intervention in:

- selected clinical parameters,
- BMI and body composition,
- nutritional status,
- resting metabolic rate and respiratory quotient,
- quality of life,
- faecal microbiota abundance and diversity,
- faecal SCFA profile and concentration,
- level of blood inflammatory cytokines.
- expression of genes of inflammatory response in skin,
- level of blood and faecal biomarkers of intestinal barrier permeability;
- concentration of urine and faecal volatile organic compounds (VOCs).
- oxidative stress parameters.

### Timeline of the study

**Fig. 2:**
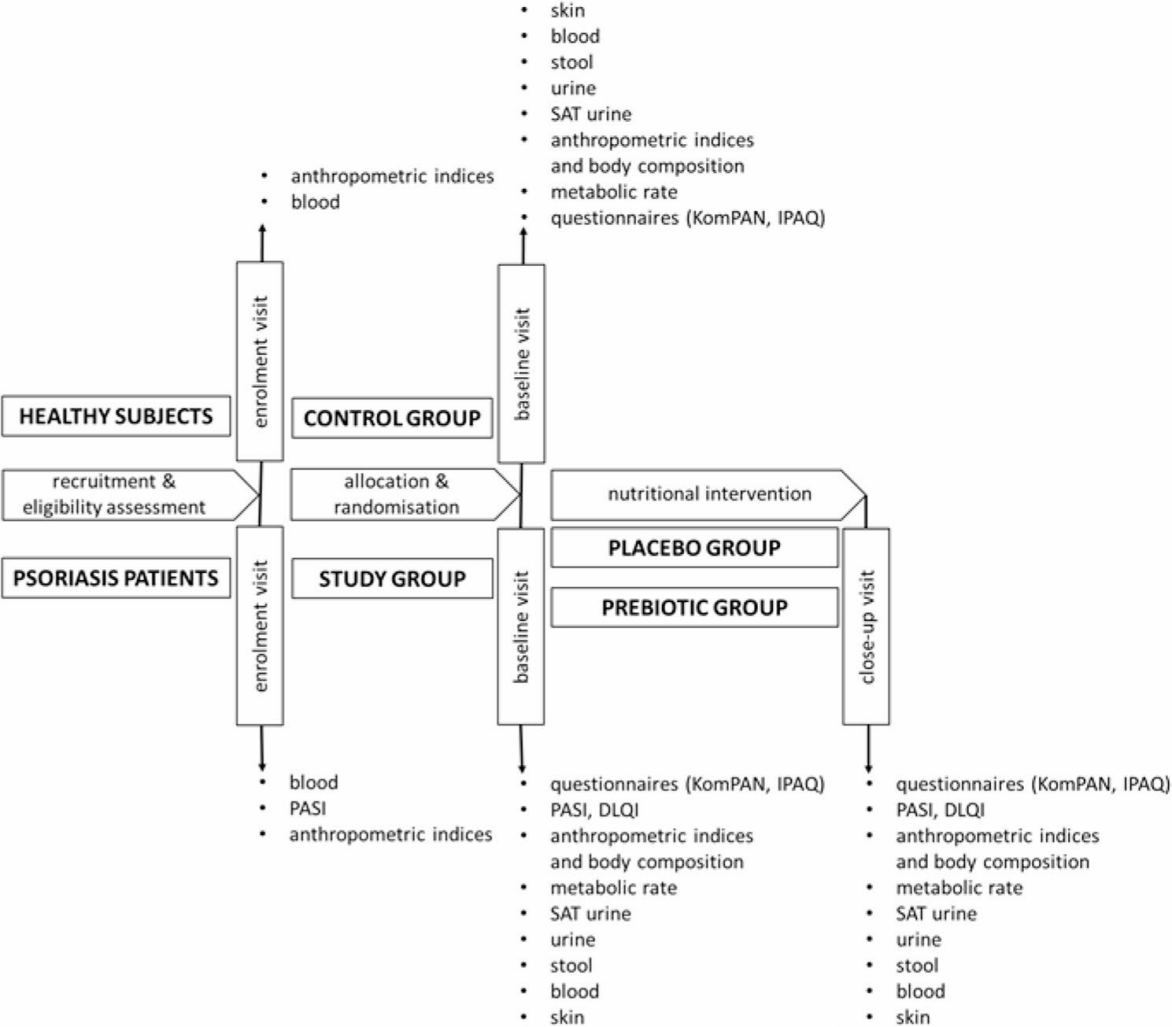
Schematic representation of study design and sampling

An overview of the study design, including the timeline of participant visits, assessment point times, and examined variables, is given using the SPIRIT diagram (Table 2) and Figs. 1 and 2.

Three sample collection points for each PS participant are planned: at enrolment (-T_1_), baseline (T_0_) and at close-up visit (T_1_), after the 8-week nutritional intervention, and two sample collection points for each healthy participant: at enrolment (-T1) and baseline (T_0_) (Table 2). Check-up visits will be carried out in the Dermatology Clinic of the Municipal Hospital in Olsztyn under the supervision of dermatologists.

**Table 2:**
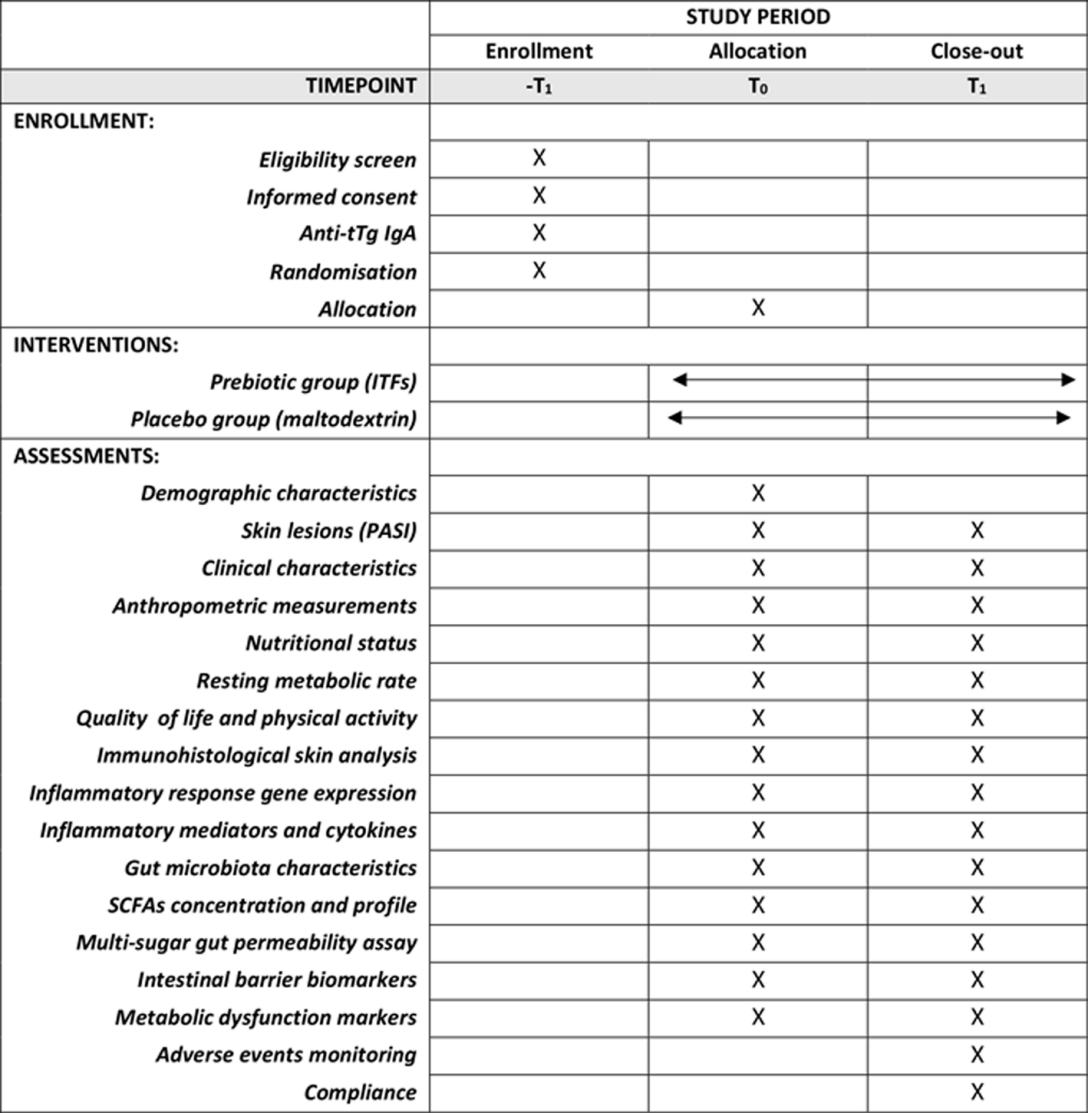
Schedule of enrolment, intervention and assessments [24]

During the enrolment visit (-T_1_), vitals (systolic, diastolic and pulse pressure), body height (stadiometer Seca 213, Seca GmbH & Co, Hamburg, Germany), and body weight (balance Seca 813 Med, Seca GMBH & Co, Hamburg, Germany) were measured in all participants. The body mass index (BMI) was calculated as a ratio of body mass to the square of body height and expressed in kg/m^2^. In addition, a qualified nurse collected a sample (8 mL) of venous blood into a serum vacuum tube (Greiner Bio-One GmbH, Kremsmünster, Austria) to perform a serum IgA antibody against tissue transglutaminase (anti-tTG IgA) assay to rule out gluten-related diseases (e.g., celiac disease) [30]. A dermatologist assessed the severity of skin lesions in PS patients using the Psoriasis Area and Severity Index (PASI).

During the check-up visits T_0_ and T_1_, vitals, anthropometric indices, and body composition will be measured, and an oral glucose tolerance test and metabolic rate evaluation will be performed in all participants after an overnight fast. Additionally, surveys on nutritional status and quality of life will be conducted, and samples of biological materials (blood, urine, urine after SAT, stool, skin) will be collected.

### Multi-sugar absorption test

Before the check-up visits T_0_ and T_1_, all participants will complete the multi-sugar absorption test (SAT) [31]. They will be instructed to perform the test on a day off from work, one day before the scheduled check-up visits. Participants will be asked to follow pre-test restrictions: maintaining normal daily activities and typical dietary patterns, but 2 days before the test, avoiding consumption of sugars (sucrose, lactulose, sucralose, erythritol and rhamnose) present in the sugar mixture, as well as alcohol, caffeine, and spicy foods.

On the test day, after an overnight fast and morning bladder emptying, subjects will ingest a sugar mixture (containing 1 g sucrose (PA-01-0238-O, POL-AURA, Morąg, Poland), 1 g lactulose (PA-03-9831-C, POL-AURA, Poland), 1 g sucralose (PA-03-9213-C, POL-AURA, Poland), 1 g erythritol (E968, Hortimex Plus Sp. z o.o. Sp. K,. Konin, Poland) and 0.5 g L-rhamnose (PA-03-1712-C, POL-AURA, Poland)), freshly dissolved in 150 mL of tap water. They will then be instructed to collect urine in a tulip-type container over 12 h. During the test, they will be permitted to drink water (150–200 mL per hour) to maintain adequate urinary output, but will not be allowed to eat for the first 5 h. Participants will collect urine samples at three intervals: 2, 5, and 12 h after ingesting the sugar mixture, and record the urine volume at each interval.

### Biological material

To ensure the appropriate amount of samples, the collection of three vials of fasting venous blood (T_0_ and T_1_) is planned from each participant: (1) 1.6 mL into a vacuum EDTA-coated tube for hematologic analysis; (2) 5 mL into a vacuum heparin-coated tube for biochemical serum analyses run by the Diagnostic Laboratory of the Municipal Hospital in Olsztyn; (3) 5 mL into a vacuum heparin-coated tube for serum analyses run at the IAR&FR PAS. The heparin-coated tubes with blood will be centrifuged at 3500 rpm for 10 min. The serum will be aliquoted and stored in Eppendorf tubes in an ultra-freezer (-80 ˚C) until further analyses (inflammatory cytokines, ELISA gut permeability biomarkers, oxidative stress parameters).

Fresh morning urine (T_0_ and T_1_) will be collected from each participant, divided into aliquots, and stored either at 4 ˚C until basic clinical assessment or in an ultra-freezer (-80 ˚C) until further analysis (VOCs, oxidative stress parameters).

Urine samples after home-made SAT will be gathered from participants before visits (T_0_ and T_1_) and transported to the IAR&FR PAS, where samples will be stored in an ultra-freezer (-80˚C) until further analysis.

Stool samples (T_0_ and T_1_) will be gathered by participants not later than 3 days before visits, kept in a domestic freezer (-20 °C), and transported frozen to the IAR&FR PAS, where samples will be divided into approximately 0.2-gram and 1-grem aliquots without defrosting, and stored in an ultra-freezer (-80˚C) until further analysis (SCFAs, VOCs and ELISA gut permeability biomarkers).

Skin biopsy specimens (T_0_ and T_1_) from PS participants will be collected from a psoriatic lesion area (different regions of lesion occurrence) and a non-lesion area (right lower quadrant of the abdomen), while from healthy individuals, a skin specimen will be collected at the right lower quadrant of the abdomen. The 4-mm punch biopsy will be performed using local anaesthesia (1% lignocaine). One biopsy specimen from each area will immediately be frozen in liquid nitrogen, then stored at -80 °C until further inflammatory response gene expression analysis; the second biopsy specimen of each area will be stored in paraformaldehyde until further immunohistological analysis.

### Clinical characteristics

The plasma and serum specimens will be aliquoted. Complete blood count, selected biochemical blood parameters (creatinine, aspartate aminotransferase - AST, alanine aminotransferase - ALT, C-reactive protein - CRP), lipid profile (total cholesterol, HDL, LDL, triglycerides, and apolipoprotein A and B), fasting glucose and insulin, and glycated hemoglobin will be determined according to standard procedures in the outpatient clinic of the Municipal Hospital in Olsztyn (Poland).

In addition, participants will undergo an oral glucose tolerance test under specific conditions (overnight fasting, no vigorous physical activity, no alcohol consumption, and no taking pain medications). They will consume a 75-gram glucose dose solution dissolved in water. The capillary blood glucose (drawn from the fingertip with single-use lancets (Accucheck Safe, Roche Diabetes Care, Poland) will be tested at 0 (fasting glucose), 15, 30, 45, 60, 90, and 120 min after drinking the glucose solution using glucometers and diagnostic strips (Contour Plus Ascensia, Ascensia Diabetes Care, Poland).

A basic urine evaluation will be conducted according to standard procedure in the diagnostic laboratory of the Municipal Hospital in Olsztyn (Poland).

### Anthropometric indices and body composition

Before anthropometric indices determination, participants will be asked to empty their bladders, change into light clothing and take off their shoes to promote consistency between each measurement. Body height will be measured on a standard stadiometer (Seca, Hamburg, Germany). The body weight and composition will be assessed by bioelectrical impedance analysis (Tanita BC-418 analyser, Tanita Corporation, Tokyo, Japan). Parameters will be estimated using the standard prediction equations and software provided by the manufacturer. The waist (at the narrowest point) and hips (around the widest part) circumference will be measured by flexible measurement tape, and the waist-hip ratio (WHR) will be calculated by dividing the waist circumference by the hip circumference.

### Nutritional status

The nutritional status of study participants will be evaluated using a validated KomPAN questionnaire and a 3-day food record recall. The questionnaires will be filled out by participants using the paper-and-pencil method to avoid the interviewer’s influence.

### Metabolic rate

The resting energy expenditure will be measured by using indirect respirometry with a ventilated mask Cortex MetaMax 3B mobile device, in conditions of thermal comfort, under fasting conditions, and in the supine position. Ventilated mobile Breath-by-Breath systems comprise components to collect and mix expired air, measure flow rate, analyse gas concentrations (oxygen was analysed using paramagnetic analysers and carbon dioxide using infrared analysers), and pump air through the system. The test will be performed after the device has been calibrated. The measurement is performed for approximately 20 min. Determining the volume of oxygen and carbon dioxide enables the determination of respiratory quotients and the degree of utilisation of fat, carbohydrates and proteins in the body.

### Determination of the quality of life and physical activity

The quality of life will be assessed using the Quality of Life SF-36 questionnaire – the Polish version or the World Health Organisation Quality of Life Instrument-WHOQOL-BREF standardised questionnaire. Additionally, in PS participants, the Dermatology Life Quality Index (DLQI) will be assessed. The full Polish version of the International Physical Activity Questionnaire (IPAQ) will be used to determine participants’ physical activity.

### Immunohistological skin analysis

In each skin specimen from lesional and non-lesional areas from PS participants and skin specimens from healthy individuals, the level of tumour necrosis factor (TNF), interferon gamma (IFN-γ), interleukins (Il-17 A, Il-17 F, Il-12, Il-23, Il-8, IL-22, Il-35) (only in affected specimens) and the profile of γδ T cells (CD3, TCRγδ CD27, CD45RA, CCR6, IL-23R), subtype Vγ9Vδ2 (in both lesional and non-lesional skin specimens) will be determined.

### Expression of inflammatory response genes

RNA isolation and gene expression (real-time PCR): Total RNA will be extracted from skin biopsy specimens using commercially available kits for RNA isolation (Rneasy Fibrous Tissue Mini Kit, QIAGEN, Hilden, Germany). Complementary DNA (cDNA) will be synthesised from mRNA using the High-Capacity cDNA Reverse Transcription (Applied Biosystems by Thermo Fisher Scientific). Before reverse transcription, samples will be treated with DNase I (Life Technologies by Thermo Fisher Scientific). The expression of specific mRNA will be quantified with TaqMan Gene Expression Assays (Applied Biosystems by Thermo Fisher Scientific) on the 7900 Real-time PCR System or an ABI ViiA™ 7-Sequence Detection System (Applied Biosystems by Thermo Fisher Scientific). All the results will be normalised to housekeeping gene expression as an endogenous control. The real-time PCR will be performed in the Molecular Biology Laboratory in the Institute. This Core Facility provides the service and equipment required for gene expression analysis (7900 Real-time PCR System and ABI ViiA™ 7-Sequence Detection System (Applied Biosystems by Thermo Fisher Scientific).

### Inflammatory mediators and cytokines

The profile of cytokines in blood serum will be determined using a commercial Bio-Plex 200 array system (Bio-Rad). A wide range of cytokines (FGF basic, eotaxin, G-CSF, GM-CSF, IFN-γ, IL-1β, IL-1ra, IL-2, IL-4, IL-5, IL-6, IL-7, IL-8, IL-9, IL-10, IL-12 (p70), IL-13, IL-15, IL-17 A, ) and chemokines (IP-10, MCP-1 (MCAF), MIP-1α, MIP-1β, PDGF-BB, RANTES, TNF-α, VEGF) will be analyzed using dedicated assay kits (Bio-Plex Pro Human Cytokine 27-plex Assay, Bio-Rad Laboratories, Hercules, CA, USA). A fully integrated system will be able to provide accurate and reproducible assay results and comprises a 96-well fluorescent microplate reader with a dual-laser detector, software and validation, and calibration kits and assays. All measurements will be repeated in duplicate.

### Gut microbiota characteristics

The hyper-variable regions of the 16S rRNA bacterial gene (V3-V8) will be amplified with primers 337F 5’- GACTCCTACGGGAGGCWGCAG − 3’ and R1391 5’- GACGGGCGGTGWGTRCA − 3’. Bacterial libraries will be created using a Ligation Sequencing Kit 1D plus either Native Barcoding Expansion or a set of custom barcodes. Final libraries will be sequenced on a GridION X5 sequencer (Oxford Nanopore Technologies, Oxford, UK). All bioinformatic analyses will be performed on the Galaxy Europe platform using publicly available, version-controlled tools. The reads obtained will be filtered for their quality with fastp (v1.0.1 + galaxy2; average read quality ≥ 9 Phred quality score ) and length (minimum 800 bp). During the demultiplexing step, the removal of adapters (crucial for preparing the reads for alignment to a reference genome, as adapter sequences can interfere with alignment accuracy) and barcodes (to ensure that only the actual RNA sequences of interest are retained for each sample, preventing sample contamination) will be performed with Porechop (v0.2.4 + galaxy1). High-quality reads will be processed for taxonomic identification at the genus level by matching the NGS sequences with sequences deposited in the NCBI using Kraken2 (v2.1.3 + galaxy2) and Bracken (v3.1 + galaxy0). Compositional analyses will be performed using total-sum scaling or relative abundances, and multivariate statistics will be applied after centred log-ratio transformation to normalise the data. Alpha diversity will be assessed using the Shannon and Simpson indices, and beta diversity will be evaluated with Bray–Curtis dissimilarity, each with Krakentools (v1.2.1 + galaxy0). Finally, differential abundance will be tested with ANCOM-BC (v1.4.0 + galaxy0) and ALDEx2 (v1.26.0 + galaxy0).

### Concentration and profile of SCFAs

The concentration and profile of SCFAs in stool samples collected from participants will be analysed using gas chromatography (Agilent 7890 A, Agilent, Wilmington, DE, USA) with a flame-ionisation detector (FID) and a 7683B autosampler, after diethyl ether extraction [32]. External standards of acetic, propionic, butyric, isobutyric, valeric, and isovaleric acids will be applied, as well as 2-ethylbutyric acid as an internal standard. Analysis will be performed in triplicate.

### Functional multi-sugar gut permeability assessment

To assess whole gut permeability (gastrointestinal and colonic permeability), urine SAT samples will be collected from participants [31]. To assess small intestine permeability, the concentration of lactulose, rhamnose, and lactulose/rhamnose (L/R) ratio will be determined; to assess colonic permeability, the concentration of sucralose, erythritol, and sucralose/erythritol (S/E) ratio will be determined in urine samples with a high performance liquid chromatography (HPLC) gradient system (LC-10, Shimadzu, Japan) coupled with a refractive index detector (RID-6 A, Shimadzu, Japan). Urine samples will be thawed and diluted with deionised water, and ion-exchange resin will be added to eliminate sodium ions, then the samples will be centrifuged. If necessary, a filtration step will be carried out to remove proteins. Sugars will be identified and quantified based on the comparison of their retention time with the external standards. Freshly prepared standards of lactulose, mannitol, sucralose, and erythritol (in ACN/H_2_O, Sigma–Aldrich) and their calibration solutions will be applied during analysis. All measurements will be repeated in triplicate.

### Intestinal barrier permeability and integrity biomarkers

Selected intestinal barrier permeability and integrity biomarkers will be analysed using commercial ELISA kits in blood and stool samples. Before each analysis, the biological material will be thawed and pre-treated according to the procedures indicated in the ELISA kits manuals. In particular, before the extraction, stool samples will be mechanically homogenised.

Analysis in blood serum: zonulin, iFABP, claudin-3, bacterial lipopolysaccharides (LPS).

Analysis in stool samples: calprotectin, β-defensin-2, α1-Antitrypsin, sIgA. The planned research will be carried out using a microplate reader (Infinite M1000 PRO, Tecan, Austria).

### Metabolic dysfunction markers

The oxidative stress parameters will be determined in blood serum samples. The oxidative status of lipids will be analysed using assays of superoxide dismutase (SOD) activity, catalase activity (CAT), and photochemiluminescence for lipid-soluble antioxidants (PCL-ACL), whereas parameters of protein oxidation will be established according to reduced and oxidised glutathione (GSH and GSSG), and status of protein glycation marker- methylglyoxal (MGO) and glyoxal (GO) by HPLC method in urine. The planned research will be carried out using a Microplate reader (Infinite M1000PRO, Tecan, Austria) with UV-VIS and fluorescence modes, Photochem^®^ apparatus (Analytical Jena, Jena, Germany), an HPLC system with autosampler (LC-10, Shimadzu, Japan) and a fluorescent detector (RF-10AXL, Shimadzu, Japan).

The qualitative and quantitative analysis of VOCs will be determined in urine and stool samples by headspace solid phase microextraction (HS-SPME) coupled with gas chromatography (7890 A GC, Agilent Technologies, USA) and mass spectrometry detector (5975 C VL MSD, Agilent Technologies, USA) [33]. Analysis of microbial volatile patterns in stool samples allows characterisation of the gut microbiota metabolites. The analysis will be preceded by SPME, then the collected urine and faeces will be portioned into an appropriate number of vials capped with a PTFE septum and a crimp cap. The compounds will be identified by comparing spectra in the mass spectral library of the National Institute of Standards and Technology (2005), comparing retention times, and calculating linear retention indices relative to a series of alkanes (C_7_-C_40_) and with standards of VOCs. The planned research will be carried out using a gas chromatograph (7890 A, Agilent, USA) equipped with MS and FID detectors.

### Compliance and adverse events monitoring

The PS participants will be provided with a questionnaire on compliance and adverse reactions (in Polish), and will be instructed to record the intake of the supplement, number of stool evacuations, appearance of stools, adverse reactions (gastrointestinal and other), changes in skin lesions, and any medications taken during the trial. At the end of the 8-week intervention, participants will be asked to return the completed questionnaire for compliance assessment.

### Sample size

As the primary outcome is the reduction in the extent and severity of disease determined as change in PASI score from baseline (T0) to T1 in PS patients supplemented with prebiotic ITFs compared with PS patients supplemented with placebo over the 8-week intervention, we assumed a 50% reduction in PASI (PASI50) as being a clinically meaningful improvement in PS treatment [34]. We estimated that 25 evaluable participants in the placebo and prebiotic groups are needed to detect a moderate-to-large between-group difference in PASI change (continuous outcome; standardised difference ≈ 0.8) with 80% power and a two-sided 5% significance level. Given an anticipated dropout rate of 20%, we estimate that 30 participants are required in each randomised study arm to ensure approximately 25 evaluable participants per group at T1. The required number of participants was calculated using the ClinCalc power calculation (clincalc.com) and confirmed with R software. PASI50 will be analysed as a secondary responder endpoint.

### Ethical aspects

The experimental design and procedures were approved by the Bioethics Committee of the Faculty of Medical Sciences at the University of Warmia and Mazury in Olsztyn (Resolution no. 1/2023 of 19 January 2023). The study protocol and all procedures planned in this RCT will be carried out in accordance with the ethical principles set out in the Declaration of Helsinki [35], and ethical and scientific standards of Good Clinical Practice. At the enrolment visit, participants will be informed about the aims, potential risks, and benefits of the planned research and will be asked to sign an informed consent form. Participants will be guaranteed sufficient time to read the study protocol and the informed consent form. If the contents of the documents are not understandable, the research team will clarify any doubts. Participants will be informed that their contribution to the study is voluntary and that they have the right to withdraw at any time. Participants will be assured that all personal data will be anonymised and stored under the GDPR. The study was registered at http://www.clinicaltrials.gov (registration number NCT05971992).

### Statistical analysis plan

The analysis of the results obtained in this RCT with the nutritional intervention will be carried out using the Intention-to-Treat (ITT) approach, providing fair comparisons among the experimental groups, i.e. results of the prebiotic supplemented group will be compared with the results of the placebo group. All randomised participants will be analysed according to the randomisation scheme using all available data. The primary endpoint (change in PASI from T0 to T1) will be analysed using a baseline-adjusted ANCOVA/linear regression model, with treatment group as the main predictor and adjustment for baseline PASI (T0) and the covariates used in covariate-adaptive randomisation (sex, age, BMI). The treatment effect will be reported as an adjusted mean difference with a 95% confidence interval (two-sided α = 0.05). PASI50 will be analysed as a secondary endpoint using logistic regression with the same adjustment set.

Statistical analyses and visualisations will be performed using the R software (www.r-project.org), Statistica 14 software (StatSoft, USA) or GraphPad Prism version 10.0.3 (275) for Windows, GraphPad Software, LLC (Boston, MA, USA). The data within groups will be tested for normality using the Shapiro–Wilk test. Levene’s test will be performed to assess the homogeneity of variances within groups. For secondary outcomes and cross-sectional comparisons, two- or three-group comparisons will be performed using a t-test or ANOVA; if assumptions are not met, alternative nonparametric tests will be conducted (Mann–Whitney or Kruskal–Wallis test). Results will be considered statistically significant when *p* < 0.05. Correlations between selected parameters will be tested using Pearson or Spearman correlation, with prior tests for heteroscedasticity. Logistic and linear regression will be used to estimate associations between parameters. Missing data will be summarised by group with reasons; sensitivity analyses will assess robustness to missingness. Baseline total energy intake and baseline habitual dietary fibre intake from foods (excluding the allocated supplement) will be considered in prespecified sensitivity analyses (and in microbiome association models where relevant) to account for background diet; total fibre intake including the study supplement will not be included as a covariate in the primary efficacy model. For high-dimensional outcomes (e.g., microbiome taxa, metabolomics/VOCs), false discovery rate (Benjamini–Hochberg) control will be applied.

## Discussion

To date, most clinical intervention studies on patients with psoriasis have focused solely on probiotics [36, 37]; in only a few recent intervention studies, the interest shifted towards a combination of beneficial microorganisms and prebiotic substances [38, 39]. However, considering the growing awareness of the impact of diet and lifestyle on the health of psoriasis patients, the recognition of prebiotics as a potential source of health benefits with minimal constraints even during therapy is relevant.

Prebiotics, including ITFs, are generally recognised as safe and have well-established beneficial health effects. The unique ability of prebiotics to modulate microbiota composition and shape its functions could be applied as a microbiota-mediated remedy to a diverse array of symptoms observed in dermatoses [40]. Moreover, prebiotics offer this enormous potential for health benefits, primarily safely but also cheaply. Despite the proven positive effects of prebiotics on the human body and well-being, there is a limited number of studies regarding the use of prebiotics in chronic skin inflammation [21, 41]. Moreover, due to the differences in protocols and methods applied, the obtained results, in particular related to microbial communities, are difficult to compare. No studies regarding the use of ITFs in PS were identified. Therefore, researching how ITFs-induced microbiota modulation impacts the immune system, inflammatory parameters, gut permeability and skin health in PS participants is well justified. Taking this into account, we proposed an RCT with nutritional intervention with chicory-derived ITFs as a dietary supplement for individuals with PS.

### Strengths, risks and limitations of the planned study

To the best of our knowledge, this is the first interventional study assessing the efficacy and effect of ITFs-induced modulation of gut microbiota in the context of improving the health status of PS patients. The chosen study model is the gold standard in estimating the effectiveness of interventions, and the applied randomisation and blinding eliminate the potential biases, which is why RCTs are one of the foundations of evidence-based medicine [42].

The protocol and the aims of our RCT were developed by an interdisciplinary research team. Well-trained researchers will follow all rigorous procedures established for this RCT to ensure that the research is conducted according to standards of best practice. Another strength of this RCT is that it is a prospective study with a comparable healthy subject control group. We expect that the wide spectrum of results obtained will provide new insights into the impact of ITFs on the range of physiological, functional, and health-related parameters, from changes in gut microbiota characteristics and intestinal barrier function to health-related and metabolic biomarkers of the gut-skin axis. This research may elucidate the nature of the interaction between gut microbiota and skin, providing further insights into the functioning of the gut-skin axis. Given the important physiological role of gut microbiota, our study will be a pioneer in assessing the efficacy of ITFs in restoring gut homeostasis in individuals with PS and will also determine whether ITF-induced microbiota modulation is an effective method for alleviating inflammation and reducing the severity of PS-related skin lesions. We anticipate that the findings from our study will enable the development of personalised dietary therapies involving ITFs, which would avoid the side effects associated with other conventional psoriasis treatments. It is worth emphasising that the proposed prebiotic intervention approach is safe, relatively straightforward to implement, and could enhance current management strategies for this dermatosis. Ultimately, we aim for our research to support a nutritional therapeutic approach for regulating systemic immunity by manipulating the gut microbiota. Specifically, we seek to stimulate the growth and activity of beneficial pro-health bacteria. We believe that such dietary modifications may help reduce the metabolic disorders commonly associated with the development of psoriasis.

This study presents several potential risks and limitations in its design. First, it is planned as a single-centre study with an estimated sample size. Ethically, it is important to generate proof-of-concept data before planning a larger-scale study. If the results of this RCT are promising, we will proceed with a multicentre study to validate the findings. Second, due to the rigorous selection criteria, the participant pool from a single centre is relatively small. To encourage participation, the local media will be involved in promoting the project through press releases, local radio, and television. This outreach will help to reach a wider audience and achieve the targeted sample size. Lastly, a potential issue is participant dropout in the long-term data collection. Since patients will take the study intervention supplements at home, they may lack motivation to continue with the supplementation, which could lead to incomplete implementation or failure to complete the intervention. To address this risk, investigators will contact patients via telephone or email to encourage adherence, address any concerns, and ensure that empty packages are returned to the investigators after the intervention is completed.

In conclusion, this article outlines the rationale and protocol for a single-centre, randomised, placebo-controlled trial evaluating a nutritional intervention. This study aims to understand the physiological and metabolic effects of ITF supplementation in patients with PS. The results of our RCT will identify the health benefits of ITF-induced modulation of the gut microbiota and provide the first clinical evidence regarding the efficacy of this approach in managing PS.

## Data Availability

No datasets were generated or analysed during the current study.

## Abbreviations

ALT: Alanine aminotransferase
ANOVA: One-way analysis of variance
AST: Aspartate aminotransferase
BMI: Body mass index
CONSORT: Consolidated Standards Of Reporting Trials
CRP: C-reactive protein
ELISA: Enzyme-linked immunosorbent assay
GC: Gas chromatography
MS: Mass spectrometry detector
iFABP: Intestinal fatty acid binding protein
IgA: Immunoglobulin A
IL-1a: Interleukin 1a
ITFs: Chicory-derived inulin-type fructans
LPS: Bacterial lipopolysaccharides
PCR: Polymerase chain reaction
SCFA: Short chain fatty acids
SD: Standard deviation
TNF: Tumour necrosis factor
